# Associations of 25-hydroxyvitamin D with fasting glucose, fasting insulin, dementia and depression in European elderly: the SENECA study

**DOI:** 10.1007/s00394-012-0399-0

**Published:** 2012-06-23

**Authors:** Elske M. Brouwer-Brolsma, Edith J. M. Feskens, Wilma T. Steegenga, Lisette C. P. G. M. de Groot

**Affiliations:** Division of Human Nutrition, Wageningen University, PO Box 8129, 6700 EV Wageningen, The Netherlands

**Keywords:** Vitamin D, Type 2 diabetes mellitus, Insulin sensitivity, Dementia, Depression, Cross-sectional

## Abstract

**Purpose:**

The classical consequence of vitamin D deficiency is osteomalacia, but recent insights into the function of vitamin D suggest that it may play a role in other body systems as well. The objective of this study was to examine the association between 25-hydroxyvitamin D (25(OH)D) and markers of glucose metabolism (*n* = 593), global cognitive functioning (*n* = 116) and depression (*n* = 118) in European elderly participating in the SENECA study. Moreover, we wanted to explore whether the observed associations of 25(OH)D with depression and global cognitive performance were mediated by fasting plasma glucose (FPG) levels.

**Methods:**

Cross-sectional associations between 25(OH)D and FPG, fasting plasma insulin (FPI) and homeostatic model assessment-insulin resistance (HOMA-IR), a marker of insulin resistance, were estimated from multiple regression analyses. Associations of 25(OH)D with global cognitive functioning (Mini Mental State Examination) and depression (Geriatric Depression Scale) were examined using Poisson regression.

**Results:**

An inverse association was observed between 25(OH)D and FPG (β-0.001), indicating a 1 % decrease in FPG per 10 nmol/L increase in 25(OH)D, but after full adjustment for demographic factors, lifestyle factors and calcium intake, this association was not statistically significant (*P* = 0.07). Although participants with intermediate and high serum 25(OH)D levels showed a tendency towards a lower depression score after adjustment for demographic and lifestyle factors, RR and 95 % CI: 0.73 (0.51–1.04) and 0.76 (0.52–1.11), respectively, these findings were not statistically significant.

**Conclusion:**

An inverse association of 25(OH)D with depression and FPG was observed, but this association was not statistically significant. There was no association between 25(OH)D with FPI and HOMA-IR or with global cognitive functioning. More studies are needed to further explore the possible role of vitamin D in the various body systems.

## Introduction

Worldwide, approximately 347 million people are affected by diabetes [1], mainly type 2 diabetes. Recent epidemiological studies suggest that diabetic patients are at increased risk of dementia [2] and depression [3]. The question whether the observed associations between these three ageing-related diseases are the result of shared risk factors or specific biological mechanisms, however, remains to be solved. Vitamin D deficiency is one of the postulated links [4–8].

Hypovitaminosis D is commonly observed in the elderly population. A restricted ultraviolet light exposure, low vitamin D intake and a decreased skin synthesis capacity may be related to the development of vitamin D deficiency in ageing populations. In NHANES III, Martins and colleagues observed lower 25(OH)D levels in women, persons ≥60 years and obese and diabetic participants [9]. In 1984, a study among middle-aged and elderly English men and women showed that post-prandial glucose levels were highest during the winter period [10]. Since then, evidence supporting a role for vitamin D in glucose metabolism expanded, including among others the identification of vitamin D receptors in the human pancreatic β-cell [11], the expression of 1-α-hydroxylase enzyme in the β-cell [12] and stimulation of the expression of insulin receptors by vitamin D in vitro [13]. The presence of 1-α-hydroxylase in cerebrospinal fluid and the existence of vitamin D receptors (VDRs) on various brain structures [14] support the hypothesis that vitamin D is involved in mental health.

However, while animal experiments point towards a protective effect of vitamin D with regard to the development of several age-related diseases, population-based studies have not yet provided conclusive evidence for the association with diabetes [4, 15], cognitive functioning [16–24] and depression [6–8, 25–28]. Therefore, our objective in this European multicentre cohort study was to examine 25(OH)D and the association with markers of glucose metabolism, cognitive functioning and depression in elderly men and women.

## Methods

### Subjects

**Fig. 1:**
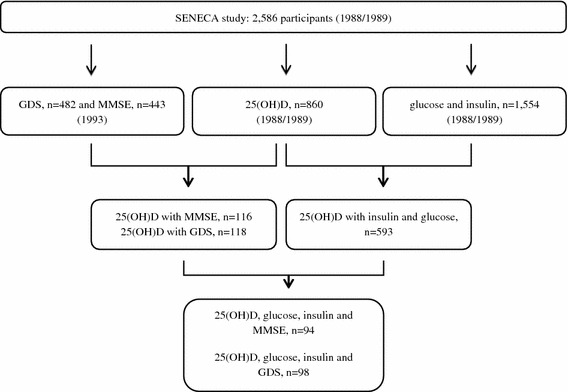
Flow diagram describing the population used in the analyses

The study is conducted using baseline data collected from European elderly participating in the SENECA study; Survey in Europe on Nutrition and the Elderly, a Concerted Action [29]. Men and women aged 70–75 years who were living in pre selected towns across Europe were invited for participation in the study. Towns were selected based upon their representativeness of the population and socio-economic structure for the whole country. Psycho-geriatric patients living in nursing homes, persons who were not fluent in the country’s language or not able to independently answer questions were excluded from participation. The total SENECA population comprised 2,586 participants. Markers of glucose metabolism and serum 25(OH)D levels were measured in 1989 and available for 1,554 and 860 participants, respectively. Of 593 participants, both 25(OH)D and markers of glucose metabolism were measured. Data on cognitive functioning and depression were obtained in 1993 and available for 443 and 482 participants, of whom serum 25(OH)D levels were measured in 116 and 118 participants, respectively. Measurements of serum 25(OH)D, markers of glucose metabolism and mental health were available of 98 (GDS) and 94 (MMSE) participants (Fig. 1). Included in the analyses were participants from: Belgium: Hamme; Denmark: Roskilde; France: Strassbourg and Valence; Hungary: Monor; the Netherlands: Culemborg; Norway: Elverum; Switzerland: Yverdon, Burgdorf, and Bellinzona; Greece: Athens and Iraklion; Portugal: Lisbon; Spain: Madrid.

#### Mental status

Global cognitive functioning was assessed using the mini mental state examination (MMSE). The variable for analyses was defined as the maximum MMSE score minus the MMSE score of the participant, reflecting the number of erroneous answers. Mitchell et al. [30] recently reviewed the accuracy of the MMSE and showed a 85.1 % sensitivity and a 85.5 % specificity in non-clinical community settings. The 15-item geriatric depression scale (GDS) was used as a screenings tool for depression. Validation with the diagnostic and statistical manual of mental disorders (DSM-IV) revealed that 97.0 % of the persons with depression (sensitivity) and 54.8 % persons without depression (specificity) were correctly classified [31].

#### Biochemical analyses

Blood samples were taken between 7.30 and 9.30 AM while the participant was in a fasting state. Fasting blood samples (25 mL) were collected and 10 mL was transferred to another tube for serum separation. A portion (0–5 mL) of each serum sample was stored at −80 °C for 25(OH)D determination. All blood samples were sent on dry ice to the coordinating centre in Wageningen, the Netherlands, and stored at −80 °C. Serum 25(OH)D values were analysed by competitive protein-binding assay (coefficients of variation: within-assay 4–7 %, between-assay 7–10 %) at the TNO Nutrition and Food Research Institute, Zeist, the Netherlands. As 25(OH)D concentrations may fluctuate seasonally, 25(OH)D was only determined in blood samples collected between January and March 1989. Plasma glucose concentration was measured by the hexokinase method using the Hitachi 911 auto-analyser. Plasma insulin concentration was measured by using enzyme immunoassay (Boehringer-Mannheim, Mannheim, Germany), and insulin resistance was calculated using homeostatic model assessment-insulin resistance (HOMA-IR) from glucose and insulin concentrations [32].

#### Covariates

Information on education level (illiterate, primary, secondary or higher education), smoking status (non-smokers, former smokers or current smokers) and presence of chronic disease (including among others stroke and hypertension) was collected using questionnaires. The Voorrips questionnaire, designed to assess physical activity level in elderly people, was used to obtain information on habitual physical activity [33]. Subjects were divided into 3 groups according to sex-specific tertiles: low, moderate or high physical activity level. Dietary intake was assessed by trained dietitians using the dietary history method. The method consisted of a 3-day estimated record and a frequency checklist of foods, based on the meal pattern of the country and with the previous month as a reference period. Portion sizes were checked by weighing quantities of food and household measures. Intakes of nutrients and food groups were calculated in each country using local food composition tables. Food consumption data were arranged into food groups following the EUROCODE classification system [34].

#### Statistical analyses

Population characteristics are reported as mean with standard deviation (SD) or percentages. Medians with interquartile range were used to report skewed variables. Chi-squared tests for categorical variables and one-way analysis of variance for continuous variables were performed to compare baseline characteristics over tertiles of 25(OH)D. Multiple regression analyses were performed to study the associations between 25(OH)D and fasting plasma glucose (FPG), fasting plasma insulin (FPI) and HOMA-IR as markers of insulin resistance. FPG, FPI and HOMA-IR were not normally distributed and therefore logarithmically transformed. β’s are presented as  % with 95 % CI per 1 nmol/L increase in 25(OH)D (Table 3). As both mental health variables followed a Poisson distribution, rate ratios (RRs) for 25(OH)D with global cognitive performance [35] and depression were calculated using multiple Poisson regression with the number of erroneous answers as outcome for global cognitive functioning and the number of depressive symptoms as an outcome for depression. Participants were categorized according to tertiles of 25(OH)D, using the lowest tertile as the reference category. In addition, a P- for trend across tertiles of 25(OH)D was calculated. All analyses were adjusted for age, sex (model 1), BMI, education, smoking, alcohol consumption, physical activity, study centre (model 2) and intake of calcium (model 3). To control for total energy intake, calcium intake was adjusted for total energy intake by using the regression residual method. The analyses were performed using the statistical package SAS, version 9.1 (SAS Institute Inc., Cary, NC, USA).

## Results

General characteristics of the study population are presented in Tables 1 and 2. The mean ± SD 25(OH)D level of the total population was 37.8 ± 20.6 and ranged from 6 to 141 nmol/L. Serum 25(OH)D levels below 50 nmol/L and below 75 nmol/L were observed in 79 % and 94 % of the participants, respectively. Participants living in the southern part of Europe were more likely to have a suboptimal 25(OH)D level than those living in northern countries (data not shown). Those with the highest 25(OH)D levels were more likely to be men (*P* = 0.02), older (*P* < 0.0001) and higher educated (*P* = 0.001). Moreover, BMI (*P* = 0.07), FPG (*P* = 0.04), FPI (*P* = 0.06) and HOMA-IR (*P* = 0.05) were lower, and physical activity levels (*P* = 0.0005) and total cholesterol concentrations (*P* = 0.001) were higher among those with the highest serum vitamin D levels. Thirty-two per cent of the participants had FPG levels, which exceeded 6.0 mmol/L. Mean MMSE and median GDS scores of the population were 27.4 ± 2.0 and 2.0 (IQR 3.0), respectively. As the maximum score on the MMSE and GDS is, respectively, 30 and 15, these results indicate a low prevalence of mild cognitive impairment or depressive symptoms. When compared with the total sample, persons in the mental health subsample were somewhat younger, had a lower prevalence of chronic disease and moreover parameters of glucose metabolism were slightly lower.

**Table 1:** Characteristics of 593 elderly European men and women of the SENECA study per tertile of serum 25(OH)D

	T1 (6–27 nmol/L)	T2 (28–42 nmol/L)	T3 (43–141 nmol/L)	*P* value
*N*	204	196	193	
25(OH)D (nmol/L)	19.2 ± 5.3	34.3 ± 4.1	61.0 ± 18.3	<0.0001
Men, *n* (%)	86 (42)	98 (50)	108 (56)	0.02
Age	74.9 ± 1.4	74.6 ± 1.5	74.2 ± 1.6	<0.0001
Body mass index^a^	27.4 ± 4.4	26.8 ± 4.1	26.5 ± 3.4	0.07
Fasting plasma glucose (mmol/L)	6.2 ± 1.9	6.1 ± 1.6	5.8 ± 1.5	0.04
Fasting plasma insulin (pmol/L)	67.9 (46.8)	74.5 (61.3)	62.4 (43.4)	0.06
HOMA-IR	1.31 (0.91)	1.45 (1.17)	1.21 (0.88)	0.05
Chronic disease present, *n* (%)^b^	166 (81)	158 (81)	144 (75)	0.24
Total cholesterol (mmol/L)	6.2 ± 1.2	6.5 ± 1.3	6.7 ± 1.1	0.001
Hypertension, *n* (%)	49 (24)	42 (21)	30 (16)	0.23
Stroke, *n* (%)	5 (2)	13 (7)	11 (6)	0.10
*Smoking status, n* (%)				
Non-smoking	122 (60)	106 (54)	100 (52)	0.16
Current smoker	39 (19)	33 (17)	31 (16)	
Former smoker	43 (21)	57 (29)	62 (32)	
Physical activity level, *n* (%)				
Low	44 (22)	30 (15)	16 (8)	0.0005
Average	74 (36)	67 (34)	58 (30)	
High	86 (42)	99 (51)	119 (62)	
Educational level, *n* (%)				
Primary education	122 (60)	110 (56)	102 (53)	0.001
Secondary education	48 (23)	64 (33)	54 (28)	
Higher education	14 (7)	9 (4)	29 (15)	
Illiterate	20 (10)	13 (7)	8 (4)	
Calcium intake (mg/day)^a^	960 ± 433	998 ± 424	1,021 ± 358	0.33
Alcohol intake (g/day)^a^	0 (10)	2 (13)	1 (9)	0.17

Values are expressed as a mean ± SD, median with IQR or *n* (%). *P* value for χ^2^ test for categorical variables and one-way analysis of variance for continuous variables

^a^12 missing values

^b^Presence of chronic disease was defined as hypertension, ischaemic heart disease, stroke, malignancy, arthritis/arthrosis, inflammatory bowel disease, respiratory problems, chronic liver disease, osteoporosis, Parkinson, others

**Table 2:** Mental health characteristics of 135 elderly European men and women of the SENECA study per tertile of serum 25(OH)D

	T1 (7–33 nmol/L)	T2 (34–52 nmol/L)	T3 (53–125 nmol/L)	*P* value
*N*	50	43	42	
Age	73.8 ± 1.8	73.7 ± 1.7	73.2 ± 1.6	0.06
MMSE score^a^	27.3 ± 2.1	26.8 ± 2.1	27.9 ± 1.9	0.06
GDS score^b^	2.0 (3.0)	2.0 (2.5)	2.0 (2.0)	0.32
Fasting plasma glucose (mmol/L)^c^	5.7 ± 0.9	6.1 ± 2.1	5.7 ± 1.3	0.43
Fasting plasma insulin (pmol/L)^c^	63.8 (46.3)	64.8 (57.4)	48.6 (44.3)	0.84
HOMA-IR^c^	1.17 (0.88)	1.22 (1.01)	0.93 (0.89)	0.76
Chronic disease present, *n* (%)^e^	34 (77)	26 (68)	28 (67)	0.54
Calcium intake (mg/day)^d^	899 ± 352	934 ± 325	1,064 ± 360	0.07

^a^19 missing values

^b^17 missing values

^c^21 missing values

^d^11 missing values

^e^Presence of chronic disease was defined as hypertension, ischaemic heart disease, stroke, malignancy, arthritis/arthrosis, inflammatory bowel disease, respiratory problems, chronic liver disease, osteoporosis, Parkinson, others

Table 3 presents the decline in FPG, FPI and HOMA-IR in percentages per 1 nmol/L increase in 25(OH)D. An inverse association was observed between 25(OH)D and FPG (−0.1 %, 95 % CI: −0.2, 0.0), indicating a 1 % decrease in FPG per 10 nmol/L increase in 25(OH)D; however, after adjustment for demographic factors, lifestyle factors and calcium intake, this association was not statistically significant (*P* = 0.07). Significant inverse associations were also found for 25(OH)D with FPI and HOMA-IR, but these were attenuated after adjustment for covariates. Stratified analysis for calcium intake demonstrated a stronger association for 25(OH)D with FPG for those with a high calcium intake (−0.2 %, 95 % CI: −0.3, 0.0), compared to those with a low intake (0.0 %, 95 % CI: −0.2, 0.1) (data not shown in table). The interaction term, however, was not statistically significant, *P* = 0.38 (data not shown in table).

**Table 3:** Associations between 25(OH)D and markers of glucose metabolism of 593 men and women participating in the SENECA study, presented as  % with 95 % CI per 1 nmol/L increase in 25(OH)D

	Fasting plasma glucose (mmol/L)		Fasting plasma insulin (pmol/L)		HOMA-IR	
Crude	−0.1	−0.2, 0^e^	−0.4	−0.7, 0^d^	−0.4	−0.7, 0
Model 1^a^	−0.1	−0.2, 0^e^	−0.3	−0.6, 0.1	−0.3	−0.9, 0.1
Model 2^b^	−0.1	−0.2, 0	−0.1	−0.4, 0.3	−0.1	−0.5, 0.3
Model 3^c^	−0.1	−0.2, 0	−0.1	−0.4, 0.3	−0.1	−0.5, 0.3

^a^Adjusted for age and sex

^b^Adjusted for age, sex, BMI, education (categorical), alcohol intake (categorical), smoking (categorical), physical activity (categorical) and study centre (categorical)

^c^Adjusted for age, sex, BMI, education (categorical), alcohol intake (categorical), smoking (categorical), physical activity (categorical), study centre (categorical) and calcium intake (continuous)

^d^ *P* < 0.05

^e^ *P* ≤ 0.01

Data on serum 25(OH)D levels and depression were available of 118 participants (Table 4). Fully adjusted models showed that compared to the reference group, those in the middle or upper tertile of 25(OH)D had on average a 27 % (RR 0.73, 95 % CI: 0.51–1.04) and 24 % (RR 0.76, 95 % CI: 0.52–1.11) (*P* for trend: 0.16) lower depression score, respectively. Additional adjustment for calcium intake (RR upper tertile 0.82 and 95 % CI: 0.59–1.14), FPG (RR upper tertile 0.88 and 95 % CI: 0.61–1.25) (*n* = 83, data not shown in table) or the prevalence of hypertension (RR upper tertile 0.82 and 95 % CI: 0.59–1.14, data not shown in table) did not alter the direction of the results.

**Table 4:** Associations between 25(OH)D and mental health of 118 men and women participating in the SENECA study

	T1 (0–34 nmol/L)	T2 (34–52 nmol/L)	T3 (52–125 nmol/L)	*P for trend*
*GDS (depression)*				
Crude model, *n* = *118*	1.0	0.78 (0.53–1.14)	0.76 (0.50–1.15)	0.05
Model 1^a^, *n* = *118*	1.0	0.80 (0.55–1.16)	0.76 (0.49–1.17)	0.05
Model 2^b^, *n* = *103*	1.0	0.73 (0.51–1.04)	0.76 (0.52–1.11)	0.16
Model 3^c^, *n* = *103*	1.0	0.74 (0.53–1.06)	0.82 (0.59–1.14)	0.41
*MMSE (global cognitive functioning)*				
Crude model, *n* = *116*	1.0	1.19 (0.87–1.64)	0.78 (0.54–1.12)	0.04
Model 1^a^, *n* = *116*	1.0	1.19 (0.86–1.63)	0.76 (0.54–1.08)	0.04
Model 2^b^, *n* = *103*	1.0	1.42 (1.02–1.97)^d^	0.92 (0.63–1.36)	0.39
Model 3^c^, *n* = *103*	1.0	1.39 (1.00–1.94)^d^	0.94 (0.63–1.39)	0.51

^a^Adjusted for age and sex

^b^Adjusted for age, sex, BMI, education (categorical), smoking (categorical), physical activity (categorical), alcohol intake (categorical) and study centre (categorical)

^c^Adjusted for age, sex, BMI, education (categorical), smoking (categorical), physical activity (categorical), alcohol intake (categorical), study centre (categorical) and calcium intake (continuous)

^d^ *P* ≤ 0.05

Among 116 participants of whom 25(OH)D concentrations were known and the MMSE was completed (Table 4), age- and sex-adjusted models did not show significant associations for those in the middle or highest vitamin D group, RR 1.19 (95 % CI: 0.86–1.63) and RR 0.76 (95 % CI: 0.54–1.08), respectively. Further adjustment unexpectedly resulted in a statistically significant higher number of erroneous answers for those with intermediate vitamin D levels, RR 1.39 (95 % CI: 1.00–1.94). No such association was however observed for those with the highest vitamin D levels, RR 0.94 (95 % CI: 0.63–1.39). Associations did not substantially change when FPG levels, hypertension or depression were included in the model (RRs upper tertile 0.90 (95 % CI: 0.56–1.45), 1.03 (95 % CI: 0.69–1.55) and 0.89 (95 % CI: 0.58–1.35), respectively, data not shown in table).

## Discussion

In this cross-sectional population-based study among European elderly, participants with higher serum 25(OH)D concentrations tended to have less depressive symptoms. The data does not support the hypothesis that higher serum vitamin D levels are associated with a better cognitive performance. Moreover, despite a modest inverse association between 25(OH)D and fasting plasma glucose, the hypothesized independent health benefits of 25(OH)D on insulin resistance could not be confirmed in this study.

Before interpreting the results, several methodological issues warrant further discussion. First of all, blood samples were collected during the winter season and therefore reflect the lowest 25(OH)D concentrations throughout the year. Secondly, serum 25(OH)D was measured only once and may therefore not reflect long-term status. Furthermore, the debate on the most accurate method to determine serum 25(OH)D levels is still ongoing [36], but the competitive protein-binding (CPB) assay applied in this study might not be the most optimal method. A previous study comparing different serum 25(OH)D assays showed that CPB assay was highly correlated with radioimmunoassay (RIA) (*r* = 0.72) and high-performance liquid chromatography (HPLC) (*r* = 0.69). Additionally, mean 25(OH)D levels as measured with CPB assay appeared to be systematically higher compared to HPLC and RIA [37]. The CPB assay used in our study may therefore have resulted in an overestimation of the true 25(OH)D status. However, since it concerns a systematic overestimation, it will not have affected the strength or the direction of the observed associations. A strength of this study is that 25(OH)D samples were taken in ten countries all over Europe, collected during the same month and analysed in one single laboratory. Therefore, seasonal variation or inter-laboratory variation cannot have affected the results. Another strength of the SENECA database is that it includes extensive information on lifestyle and dietary factors including physical activity level and calcium intake, reducing the possibility of confounding significantly. Residual confounding by potential covariates as PTH, however, cannot be ruled out.

Descriptive analyses showed lower 25(OH)D levels among those with a higher BMI, lower physical activity level and persons at older age. It may be suggested that in our population, those with a higher physical activity level performed part of their exercises outdoors, which may have resulted in higher 25(OH)D levels. The decrease in 25(OH)D with age may be explained by the fact that the production of vitamin D in the skin decreases while ageing [38]. Lower 25(OH)D levels among persons with a higher BMI have also been observed in previous studies and have been suggested to be the consequence of the storage of 25(OH)D in fat tissue and thus not being bioavailable in serum [39].

Among individuals in the SENECA study, FPG decreased with 1 % when 25(OH)D increased with 10 nmol/L; however, this was not statistically significant after adjustment for multiple factors. No association was observed between 25(OH)D with FPI or HOMA-IR. Biological evidence that vitamin D may affect markers of glucose metabolism and the development of diabetes is increasing [4, 40]. Previous published cross-sectional studies show however inconsistent results [41–46]. Among 142 Dutch men aged 70–88 years, 25(OH)D was inversely associated with the area under the curve for both glucose and insulin, which remained significant after adjustment for BMI, skinfold thickness, alcohol, smoking and physical activity [41]. High levels of 25(OH)D were furthermore positively associated with insulin sensitivity and inversely correlated with β-cell function in a population of 126 young adults who underwent a hyperglycaemic clamp experiment [44]. Moreover, inverse associations with measures of insulin resistance were observed among participants of the Framingham Heart Study [43] and NHANES III [46]. The LIPGENE study [45] and the Women’s Health Initiative [42] were not able to confirm previous evidence for a possible link between 25(OH)D and glucose metabolism. A recently published systematic review and meta-analyses with data of 5 prospective cohort studies showed that persons with 25(OH)D levels higher than 62.5 nmol/L had a 43 % lower risk of developing type 2 diabetes when compared to those with levels below 35 nmol/L [15]. Mitri and colleagues (2011) also summarized the results of RCTs in this field and concluded that these trials do not yet provide definite evidence for a beneficial role of vitamin D supplementation on glycemic outcomes [15].

Despite a small sample size, this cross-sectional study showed a modest non-significant association between 25(OH)D and depression. Up to now, only very few population-based studies examined the possible link between 25(OH)D and depressive symptoms, showing contradictory results. For instance, 1-year follow-up of 7,358 middle-aged and elderly Americans diagnosed with a cardiovascular event, without previous depressive episode, showed that patients with a 25(OH)D level >125 nmol/L were less often depressed compared to persons with 25(OH)D levels ≤37.5 nmol/L, HR 2.70 (1.35–5.40) [7]. Physical activity, socio-economic status or BMI were however not included as covariates. At 6 year of follow-up, women participating in the InCHANTI Study with the lowest 25(OH)D levels reported significantly more depressive symptoms, compared to those in the highest tertile. It has to be mentioned that a relatively large number (42 %) of the women participating in this study reported to have a depressed mood [8]. Furthermore, significantly lower 25(OH)D levels were also observed among 1,282 Dutch middle-aged and elderly with minor and major depressive symptoms, compared to those without depressive symptoms [6]. No clear beneficial role for vitamin D in depression was observed in population-based studies among Chinese elderly and Japanese municipal officials aged 21–67 years [25–27]. In one of these studies among Chinese elderly, a significant association between 25(OH)D and depression was observed at baseline, but not after 4 years of follow-up. The incidence rate of depression at 4 years of follow-up was 4 % and in addition only 6 % of the men had 25(OH)D levels below 50 nmol/L, which may perhaps partially explain the lack of the association observed after 4 years [25]. Inconsistencies between studies may also be the result of differences in the ascertainment and prevalence of depression, lack of adjustment for covariates and differences in geographical location. As persons with depressive symptoms may be less likely to go outside, associations between serum vitamin D and depression observed in cross-sectional studies may also be the result of reverse causation. By performing RCTs, the possibility of reverse causation can be eliminated. However, up to now, only very few trials studied the effect of vitamin D supplementation on mood or depression, showing conflicting findings [47–51].

While there was evidence of an inverse association between 25(OH)D and the number of depressive symptoms, no association was observed between 25(OH)D and the number of erroneous answers on the MMSE. Results of several small studies reviewed by Annweiler and colleagues [5], and more recent and larger community-based cohort studies show either no or a positive association between vitamin D and global cognitive performance [16, 18, 21, 23–25]. In 1,766 persons ≥65 years, low vitamin D levels appeared to increase the probability of experiencing cognitive impairment, particularly in men [21]. Results of the EPIDOS study point towards the same direction in a population of elderly women (OR 1.99, 95 % CI: 1.13–3.52, *P* = 0.02), even after adjustment for iPTH, serum calcium and depression [16]. Prospective data of the InCHANTI Study revealed that non-demented vitamin D deficient (<25 mmol/L) men and women were 64 % more likely to experience cognitive decline, compared to those in the sufficient group (≥75 mmol/l) [23]. The MrOS study [24], NAME study [18] and Os study [25] did not observe an association between serum vitamin D and global cognitive function. Recently, the first RCT on vitamin D supplementation and cognitive functioning was published, which did not show an effect of a 6-week treatment with 125 μg cholecalciferol on working memory, response inhibition or cognitive flexibility in young adults [47]. Despite the fact that we could adjust for a large number of important confounders, our sample size may not have been large enough to detect an association. Moreover, data on global cognitive functioning were collected 4 years following the baseline measurements, which may also have affected the association studied. Although serum vitamin D levels have been shown to decrease with age [52], 2-year follow-up data of 80 fragile elderly, with a mean age of 82.1 years, showed however only a 6 nmol/L decrease in 25(OH)D [53]. Therefore, we expect that only a subtle decrease in 25(OH)D levels may have occurred during the period until the mental health indicators were measured.

In conclusion, this study showed a tendency towards an inverse association of 25(OH)D with FPG and depression but not with FPI, HOMA-IR and not with global cognitive performance. As the overall evidence for a role of vitamin D in glucose metabolism and mental health is still inconclusive, more prospective epidemiological studies, meta-analysis, randomized controlled trials and mechanistic studies are warranted.
